# The systemic effects of the enriched environment on the conditioned fear reaction

**DOI:** 10.3389/fnbeh.2023.1227575

**Published:** 2023-08-22

**Authors:** Grigory A. Grigoryan

**Affiliations:** The Laboratory of Conditioned Reflexes and Physiology of Emotions, Institute of Higher Nervous Activity and Neurophysiology, Russian Academy of Sciences, Moscow, Russia

**Keywords:** enriched environment, conditioned fear reaction, context, memory, information processing, motivation, reinforcement

## Abstract

In this review, a hypothesis is proposed to explain the beneficial effect of an enriched environment (EE) on the conditioned fear reaction (CFR) from the perspective of a functional system of behavioral control. According to the hypothesis, the EE affects all behavioral act components, including the processing of sensory information, memory, motivational and reinforcing systems, and motor activities, which weakens the CFR. Animals raised in the EE have effects that are comparable to those of context (CTX) and CS pre-exposures at latent inhibition. An abundance of stimuli in the EE and constant contact with them provide the formation of CS-noUS and CTX-noUS connections that later, during CFR learning, slow down and diminish fear. The EE also contributes to faster processing of information and habituation to it. As a result, many stimuli in the context lose their significance, and subjects simply ignore them. And finally, the EE affects the motivational and reinforcing brain mechanisms, induces an impairment of search activity, and worsens memory consolidation, which leads to a reduction of CFR.

## Introduction

Empirical observations on humans and experimental work on animals show that one of the beneficial factors that corrects various psycho-neurological disorders and neurodegenerative pathologies at different ages and in different sexes is long-term exposure to an enriched environment (EE). This refers to emotional (impulsive behavior, anxiety and depressive disorders), cognitive (Alzheimer’s disease), motor (Parkinson’s disease) and other brain activities. Animal experiments have shown a protective and preventive effect of EE on the development of anxiety-depressive and cognitive disorders (Laviola et al., 2008; Hannan, 2014; Guan et al., 2021; Ji et al., 2022; Xu et al., 2022; Zhang et al., 2022) caused by prenatal and neonatal stressors (e.g., bacterial intoxication, maternal separation, etc.). EE also counteracts the development of negative consequences of severe stress like post-traumatic stress disorder (Imanaka et al., 2006; Smail et al., 2020; Yu et al., 2022). It slows down the development and alleviates the course of Alzheimer’s disease (Sommerlad et al., 2019; Li et al., 2021; Liew et al., 2022; Colavitta et al., 2023), Parkinson’s disease (Jungling et al., 2017; Alarcón et al., 2023), Huntington’s disease (Spires et al., 2004; Novati et al., 2022), autism spectrum disorders (Aronoff et al., 2016) and other pathologies. The beneficial nature of the influence, the wide spectrum of action and the relative simplicity of the procedure makes EE a “miraculous remedy” for many severe and negative consequences of emotional, mental, cognitive and motor disorders. It should be noted that the molecular-cellular and biochemical mechanisms of the positive effects of EE on behavior are quite well investigated [see the reviews of Kempermann et al. (1997), Laviola et al. (2008), Grigoryan (2021), Liew et al. (2022), Alarcón et al. (2023), etc.]. They are associated with gene-environment interactions, increased neurogenesis, synaptogenesis, plastic synaptic rearrangements, growth of trophic factors, in particular BDNF and NGF, changes in a number of subunits of NMDA and AMPA receptors, cAMP/PKA signaling pathways, etc. Moreover, the effect of EE on hippocampus-dependent behavior associated with spatial learning and memory is quite well studied. Fewer works are devoted to the study of the influence of EE on behavior based on fear conditioning, or another traumatic event that leaves a long and strong emotionally negative trace in the memory (post-traumatic syndrome). In this review, we will present the literature data on this subject and try to discuss it from the standpoint of the functional behavior control system that we proposed many years ago (Grigoryan, 1990, 2006; Grigoryan and Gulyaeva, 2017). But first, let us briefly recall the main provisions of the operation of this system.

## Functional behavioral control system

**FIGURE 1:**
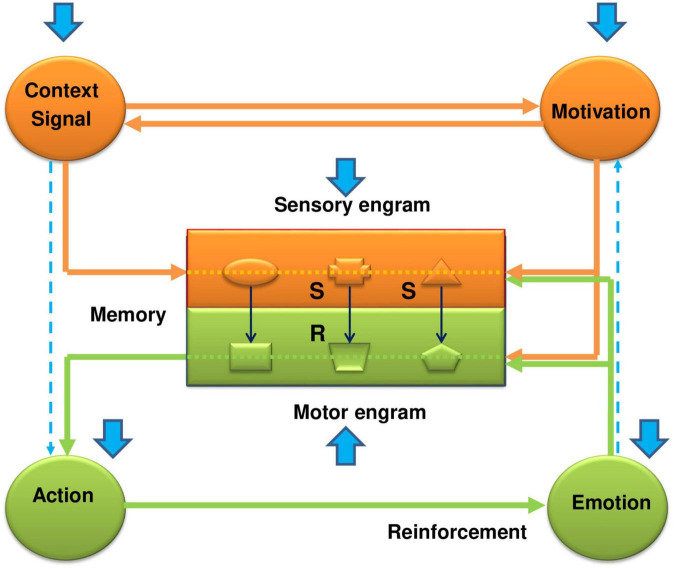
The functional system of behavioral control–how it works and how the EE has a beneficial impact on its work in the case of fear conditioning. The functional system of behavioral control represents an integrated framework of key components, including memory, sensory information, motivation, actions, and reinforcement. It consists of two main parts: sensory-motivational (orange color) and motor-reinforcing (green color) circuits. They are linked within the memory apparatus by stimulus-motor connections (S-R connections or engrams), which are organized on the complementary (“lock and key”) principle. Formation of a new stimulus-motor connection requires, on the one hand, activation of the motivational structures by a proper real stimulus via a forward conditional connection, and, on the other, formation of a trace or sensory engram in the central memory-related structures corresponding to this stimulus. It is also possible to activate the sensory-motivational subsystem from the motivational input. In this case, the motivational excitation, on the one hand, will activate the sensory engrams of different stimuli, and on the other hand, through activation of the backward conditional connection, induce the subject’s search for those stimuli that correspond to sensory engrams that led in the past to receiving reinforcement. Thus, in the sensory-motivational subsystem forming the first closed neuro-functional circuit, events develop in the following scenario: the inflow of current sensory information; trigger, with its assistance, the motivational mechanisms of the brain; actualization through signal and motivational excitations of sensory engrams of memory; comparison and evaluation of matching (or not matching) of the stored engrams with proper incoming environmental stimuli. When the sensory engrams coincide with the acting stimuli, the complementary motor engrams are actualized and excitation is transferred to the motor-reinforcement subsystem. The sensory and motor engrams form their own internal subsystem of connections in the memory apparatus, which overall are organized as a second neuro-functional circuit. The motor-reinforcement subsystem includes the executive mechanisms of actions, the mechanisms of reinforcement (emotions), and motor engrams (traces of motor experience) in the motor part of memory (shown by the green color). Overall, these functional blocks form a third neuro-functional circuit, which is closed by feedback connections from the reinforcing structures to the memory engrams. If the result of action has valuable or adaptational significance for the organism, the corresponding stimulus-motor engrams are strengthened and reinforced (Grigoryan, 1990, 2006; Grigoryan and Gulyaeva, 2017). The blue arrows show the influence of the enriched environment on manifestations of the conditioned fear response. As is seen from the figure, the EE affects every link of the functional system. At the level of the memory structures, the EE weakens the formation of conditioned (S-S) connections between the signal and the unconditional stimulus (CS-US) and between the context and the unconditional stimulus (CTX-US). This happens due to the fast adaptation of animals being in the EE to the surrounding world, which promotes an impaired incorporation of sensory stimuli into the contextual fear memory (formation of the sensory engrams). On the other hand, the quick loss of interest in these stimuli in the EE animals weakens the motivational state and, in addition to the weak impact of natural stimuli, further impairs the formation of sensory engrams. The weakening of the S-S associations under the effects of EE during fear conditioning reminds the effects of latent inhibition. Thus, the EE impairs the function of the sensory-motivational circuit and formation of the sensory engrams in the memory structures. But apart from the problems in the sensory part of the memory, the EE affects locomotor activity and reinforcement function, bringing problems with the formation of motor engrams and consolidation of fear memory. Thus, again, this all acts against the conditioned fear response, and promotes the beneficial effects of the EE. More full explanations, supported by the literature data are given in the text.

Behavioral control is carried out through self-organizing functional systems of the brain, which include the mechanisms of the main key components (categories) of an integrated behavioral act. They include the mechanisms of perception of stimuli and events of the external world, the formation of internal motivational states and needs, and the organization of movements and achievement of reinforcement (emotions). All of them are closed up in the memory apparatus, which occupies a central (integrating) place in the functional system of behavior (Figure 1). Traces of stimuli, objects and events of the external world are deposited in the memory in the form of sensory engrams, and traces of motor actions corresponding to the appropriate sensory engrams are deposited as motor engrams. In other words, the sensory engrams represent those stimuli in memory which were in the past related with reinforcement; the motor engrams represent those actions which were in the past related with reinforcement. Generally speaking, sensory engrams constitute the S-S association (memory in classical conditioning), while motor engrams compose the motor part of the S-R association (memory in instrumental conditioning).

The functional system consists of two main subsystems–sensory-motivational and motor-reinforcing. Both subsystems are interconnected with each other through the memory apparatus by means of stimulus-motor engrams, which are organized by the complementary principle (“key-lock”). The learning process is characterized by the formation of new stimulus-motor and/or stimulus-stimulus associations under the influence of current natural environmental stimuli and internal motivational states (Figure 1). In the sensory-motivational subsystem, which forms the first closed neurofunctional circle, the events are developed by the following scenario: the inflow of current sensory information; trigger, with its assistance, the motivational mechanisms of the brain; actualization through signal and motivational excitations of sensory engrams of memory; comparison and evaluation of matching (or not matching) of the stored engrams with proper incoming environmental stimuli. In short, at the level of the sensory-motivational subsystem, a comparison and evaluation of incoming current information from the outside world with that stored in memory occurs (Gray, 1982). When sensory engrams match their corresponding natural stimuli, then the motor engrams become active and trigger activation of the motor-reinforcing subsystem. In fact, sensory and motor engrams form their own internal subsystem of connections (internal circle) in the memory (stimulus–response, S–R or stimulus - stimulus, S–S associations). This subsystem is chronologically constantly updated with new associations with their various and multiple comparisons and matching with the current moment, the formation of new combinations, checking and rechecking of old combinations, etc.

The motor-reinforcing subsystem includes executive mechanisms of actions, mechanisms for evaluation of the results of actions (reinforcement, emotions) and motor engrams (the traces of past motor actions) that form the “motor-reinforcing” part of the memory apparatus (Figure 1). Together, they form the third neurofunctional circle, which closes up in memory apparatus due to feedback from the results of the action (reinforcement) to motor engrams. If the results of actions have a positive adaptive meaning for the organism, then the stimulus-motor engrams get amplification and consolidation. If the results of action do not have a positive value, then the underlying stimulus-motor engrams weaken or completely disintegrate. Thus, the two independent enclosed subsystems are integrated into one holistic functional system through the internal stimulus-motor or stimulus-stimulus associations within the memory apparatus. At the same time, the mechanisms of memory themselves are implemented through three sensory, motivational and reinforcing (emotional) inputs. Due to the first two inputs, the formation and actualization of sensory-motor engrams take place, and, thanks to the third input, their consolidation does occur.

## What is enriched environment?

Environmental enrichment has three main components. The first component is sensory enrichment (Cummins et al., 1977; Rosenzweig et al., 1978; Van Praag et al., 2000). It is associated with filling the environment with a variety of objects for sensory perception and subsequent manipulation of them. In real life, animals and humans use environmental objects for orientation in space and formation of sensory engrams in memory, which are used to search for food, water, a sexual partner, avoid an enemy, and execute other adaptive actions. With a lack of sensory stimulation, memory works and develops poorly, the adaptive actions are not performed; life becomes dull, boring and monotonous (Costa-López et al., 2021). For instance, the individuals with a loss of vision showed significant impairment in different cognitive tasks: tactile spatial memory (Vecchi and Girelli, 1998), tactile spatial attention (Forster et al., 2007), and general cognitive skills (Nejati, 2018). More than 100 years ago, I.P. Pavlov drew attention to the impotence and complete indifference of dogs to the outside world after the extirpation of the cerebral cortex where the sensory engrams are represented. This all shows how important the sensory stimulation is or the sensory component of EE for the organism. Procedurally, this component is easily achieved by placing various replaceable items in the home cages of rats and mice: toys, nest building material, polyethylene tubes, rubber pieces, cuttings for burrowing, etc. The second component of EE is social stimulation, or the need to live in a community of conspecific partners (Brenes et al., 2016). The beneficial effect of the social component is especially evident in comparison of animals and humans having been in social isolation (Mora-Gallegos and Fornaguera, 2019; Davim et al., 2021). Prolonged social isolation produces severe stress which leads to strong negative consequences for an organism, from serious diseases with impaired cognitive functions to the development of anxious and depressive disorders. Procedurally, this component is achieved by keeping rats or mice in larger groups compared to standard housing. The third component of the EE is physical activity, or the performance of specific physical actions, which are even more effective in the positive effects of EE, for example, in inducing neurogenesis and synaptic plasticity, than the other EE components (Duman et al., 2008; Campeau et al., 2010; Rabadán et al., 2019). Physical actions represent an integral part of the formation of stimulus-motor associations, which ultimately lead to the getting of reinforcement. Procedurally, this EE component is achieved by using ladders, a treadmill for running, hammocks, and other devices for motor actions. Thus, the EE activates and stimulates the key components of the entire functional system - from the perception and processing of sensory information, to the actualization of stimulus-motor or stimulus-stimulus engrams stored in the memory, and triggering motor actions for achievement of the final positive result (reinforcement). In this sense, the EE acts as a very valuable activator of the functional system that switches over the subject from a sluggish, boring condition to an active and workable state.

## Influence of enriched environment on the conditioned fear reaction

In accordance with the functional system considered above, the influence of the EE on behavior is complex and multilateral, involving simultaneously all the key components of this system. However, for convenience of presentation, we will focus on each of them separately. But first, let’s move on to a brief description of the methodology for studying conditioned fear reaction (CFR).

## Fear conditioning model

The most convenient model for studying CFR is the Pavlovian classical defensive conditioned reflex (CR), well known in the literature as fear conditioning. A pairing of a conditional (cue) stimulus, CS (for instance, a tone) with an US stimulus (electroshock) produces a CFR, which is estimated by the percentage of the freezing time followed by 24 h (retention test) in the same context (CTX) and in response to the cue stimulus (CS). During the acquisition of the CFR, several associations are formed in the brain: between CS and US, between context and US (CTX-US), and between context and cue stimulus (CTX-CS) (Grahame et al., 1994; Escobar et al., 2002). The degree and strength of these associations depend on the magnitude and number of CS and US pairings, and the loads and presentations of environmental stimuli. With regard to the effects of EE on the CFR, many factors should be taken into account, such as the duration of staying in the EE, the age of the animal from which EE is started, features of the EE procedure (for example, its application before the onset of stress or after it); the character of the assessment tests and models used (Clemenson et al., 2015; Hegde et al., 2017; Cavalcante, 2019; O’Leary et al., 2019; Cavalcante et al., 2020; Cheng S. T. et al., 2022) and so on. Of particular interest is the effect of EE on the developing brain. The use of enriched environment as a developmental intervention was investigated in laboratory animals as a modulator of developmental trajectories (Ball et al., 2019). This intervention is especially effective during critical or sensitive periods, when brain plasticity is affected by a new experience. It was shown that the EE improves synaptogenesis and the survival of neurons during early development (Van Praag et al., 2000). The EE speeds up the development of the visual system (Sale et al., 2009), affects an increase of BDNF, which promotes neuron growth and maturation, and accelerates development of the GABA system in the visual cortex of normal rat pups, which affects visual system development. It also provides the increased sensory stimulation needed to recover from age-related decline and improve cognitive abilities (Leon and Woo, 2018).

According to our hypothesis, the EE, having general beneficial (positive) effects, should modify the work of the functional system in a way to ameliorate the CFR. The weakening of conditioned fear under the influence of the EE can be carried out through various mechanisms, which will be considered below.

## Enriched environment and memory

### Acquisition of the conditioned fear reaction

Barbelivien et al. (2006) studied the effects of EE on CFR in rats under different conditions of CS-US pairings. At first, the authors trained the rats to the classical Pavlovian CR by 4-fold pairings of CS (tone, 15 s) and US (electrical shock, 0.6 mA, 0.8 s). They also used the procedure of pseudo-conditioning, in which US was also presented 4 times, but tone was not associated with US; it was applied 2 times before and 2 times after the presentation of US with an average interval between trials of 202 s. In the case of the classical fear conditioning, the contextual memory of the EE group 24 h later in the retention test was significantly improved. The percentage of freezing time was substantially higher in the EE rats in the same context compared with the standard (STAND) housed group. The cue memory of the EE group was weaker than in the STAND group. In the case of the pseudo-conditioning, on the contrary, the contextual fear memory in rats of the EE group was substantially impaired compared to the control group; the cue memory was not changed. So, it looks like there is a link between the strength of the CS-US associations and the effects of the EE. If the conditioned associations are weak or not presented at all, as in the case of pseudo-conditioning, then the EE ameliorates the contextual fear memory. And, vice versa, if the associations are strong, then the EE strengthens the contextual fear memory. Perhaps the reverse is also true, i.e., being in the EE weakens the CS-US associations compared to the standard conditions. And, vice versa, housing in a poor environment (for instance, in social isolation) makes these associations stronger. Apparently, the EE somehow modifies the processing of sensory information, and provides a specific perception and evaluation of this information relative to other environmental conditions. It is well known from the Pavlovian laboratories and western literature (Rescorla and Wagner, 1972; Mackintosh, 1975) that the formation of conditioned associations occurs as a result of a complex conditioned stimulus, which includes a specially selected cue stimulus (CS) and contextual (CTX) stimuli paired with an unconditioned stimulus (US). The magnitude and stability of the conditioned associations depend on the strength and temporal relationships of these stimuli. For establishing a stable association, not only is the value of the US important, but also the ratio of the intensity of CS and CTX. When the CS is strong, then the effects of the context are overshadowed, and vice versa, when the context is well pronounced and filled with well expressed object-stimuli, the association between CS and US becomes weaker. It seems that a long stay in the EE adapts animals to the environmental stimuli and impairs the incorporation of these stimuli (formation of sensory engrams) into contextual memory. This can occur either directly through the mechanisms of rapid habituation to the contextual stimuli, or indirectly, through the increased activity of CS. Facilitation of CFR due to increased activity of CS under the EE conditions has not been manifested in many experiments (Rampon et al., 2000; Duffy et al., 2001; Tang et al., 2001; Barbelivien et al., 2006; Pavlova et al., 2023). At the same time, a lot of data demonstrating a link between the effects of EE and contextual fear memory were obtained, although these data had contradictory results (Rampon et al., 2000; Benaroya-Milshtein et al., 2004; Hendriksen et al., 2010; Lee and Noh, 2016; Sun et al., 2016; Sukegawa et al., 2022; Yu et al., 2022). For example, the percentage of freezing time of rats housed in the EE was much less in a neutral context than in the context where animals were experienced with the paired CS-US stimulations (Barbelivien et al., 2006). These experiments were carried out twice a day for 9 days, with alternately testing rats in a neutral setting and in a context where they received paired CS-US. In a neutral environment, where the conditioned associations were weak (due to generalization of fear transferred from the “dangerous” context), the rats of the EE group expressed the conditioned fear in a less extent than the rats of the STAND group. Moreover, the freezing time in rats of the STAND group, in contrast to the animals of the EE group, was approximately the same in the neutral and “dangerous” contexts. Similar data was obtained by Hegde et al. (2017). In their experiments, the rats were trained for CFR in the context of chamber 1, and CFR was tested in the other, albeit very similar context, chamber 2. The rats housed in the EE froze significantly less time in chamber 2 compared to the rats of the STAND group, though no differences were observed between both groups in chamber 1. In our experiments (Pavlova et al., 2023), in the retention test 24 h after fear conditioning learning, the male rats of the EE group froze significantly less time in the same context and in response to CS than the rats of the STAND groups. In the females of the EE group, the freezing time in the context, but not for CS, was shorter than in the rats of the STAND group. The interesting results on mice were obtained by Yu et al. (2022). The EE groups were subjected to the enrichment procedure on days 0–17. The other groups were housed in the standard cage. On day 17, mice were placed in the footshock box for 2 min, and then subjected to footshock treatment (2 mA, 10 s). On days 18–20, all mice were given a situational reminder procedure in the same footshock box for 2 min with the cue ball but no footshock. All mice were assigned to the no EE/no cue, no EE/cue, EE/no cue, and EE/cue groups. Mice housed in the EE had much less freezing than those housed in the STAND conditions. The cue ball which was placed into the home cages for 17 days also affected the CFR, making it weaker in the EE group compared with STAND housed animals. Interestingly, the combination of EE and cue revealed the highest reduction in footshock-induced fear behavior. Apart from the fear conditioning the EE improved memory of aversive stimuli also in the passive avoidance (Leger et al., 2015) and active avoidance learning in mice (Pietropaolo et al., 2014) and also facilitated shuttle chamber avoidance responses in rats in a PTSD model (Takahashi et al., 2014; Tanichi et al., 2018).

### Extinction of the conditioned fear reaction

During the CFR extinction, the conditioned associations between CS and US become weaker. According to our hypothesis, the animals housed in the EE should extinguish the CFR faster than the animals housed in the STAND environment. This was confirmed in many experiments (Hunter, 2015; Lach et al., 2016; Hegde et al., 2017; Cavalcante et al., 2020; on others). In particular, Lach et al. (2016) showed that the extinction of CFR in context occurs faster in rats housed in the EE (2 weeks) compared to the STAND group. The percentage of freezing time in the EE group decreased from 60 to 20–25% in the 2nd extinction session and continued to decrease in the 3rd session, while in the STAND group, a significant drop in the percentage of freezing time occurred only in the 3rd session. After 4 extinction sessions, the rats were regrouped again into three groups (STAND-STAND, EE-EE, and STAND-EE). The 1st and 2nd groups were housed under the same conditions as before, but the 3rd group of animals was replaced from the STAND environment to the EE. The lowest percentage of freezing time was seen in the EE-EE group (1%), then in the STAND-EE group (2%), while in the STAND-STAND group the freezing time was much higher (12%). In the study of Yu et al. (2022), mice exposed to the EE (from birth to PND 17) showed a significant decrease of CFR in the context where they received a single CS-US pairing with a strong US (2 mA, 10 s). In three successive extinction trials (PNDs 18–20), all mice showed a progressive decrease in CFR, but the mice housed in the EE extinguished the CFR much quicker than animals of the STAND group. Tang et al. (2001) examined the effects of EE on control and transgenic (NR2B) mice with enhanced NMDA receptor function. According to the authors, the increased activities of NMDA receptors are provided by the EE effects. Of the three extinction trials, in trial 1, no differences in freezing time were seen between the groups. But in the 2nd, and especially in the 3rd trial, the freezing time of transgenic mice of the STAND group, as well as control and transgenic mice of the EE group, compared with control naive animals was significantly shorter both in the context and to presentation of CS. Cavalcante et al. (2020) found that a 2-week stay in the EE caused a significant decrease in the freezing time during extinction of CFR compared to control animals, while a 4-week stay in the EE reduced the freezing time only in the last session. In our work (Pavlova et al., 2023), the percentage of the freezing time decrease during extinction in the EE group in context was greater than in rats of the STAND group. Thus, the extinction of contextual CFR in rats and mice occurs much faster in the EE group compared to the STAND group.

### Reconsolidation of the conditioned fear reaction

Memory reconsolidation occurs when a previously consolidated memory is reactivated (recalled) by placing the animal in the same context in which the original memory was formed, or by applying a cue stimulus without reinforcement. As a result of this procedure, the memory undergoes destabilization, becomes labile and weaker than the original memory (Lewis, 1979; Nader et al., 2000; Grigoryan and Markevich, 2015). Since the conditioned association is getting weaker during the memory reconsolidation procedure, then the EE, according to our hypothesis, would have a stronger effect on fear memory reconsolidation than the STAND conditions. We were able to find only one work in the literature that studied the influence of housing conditions on the reconsolidation of CFR memory (Schroyens et al., 2023). In this work, the rats were placed in different living conditions (EE, social isolation, SI and STAND), starting from the 26th until the 68th PND. The acquisition of CFR was established in the context without application of CS (3 electrical shocks were given with an interval of 30 s). 24 h later, the contextual fear memory was reactivated by placing the animals in the same environment for 5 min without a shock. 24 h later, in the retention test (10 min) the percentage of freezing time was measured in rats of different groups. The results obtained did not confirm the prediction of the hypothesis, since no differences were seen in the freezing time of the EE and STAND groups neither during reactivation nor retention tests. At the same time, the rats that were subjected to SI froze to a less extent than animals of the other groups.

### The enriched environment and conditioned fear reaction caused by other stresses

The hypothesis predicts that the EE should counteract the increase of CFR under the influence of additional stresses, i.e., reduce the total freezing time and conditioned fear. Novaes et al. (2021) housed the animals in the EE and STAND conditions for 2 weeks and then divided each group into two subgroups, one of which was subjected to a single restrictive stress for 2 h, and the other was not subjected to it. After 10 days, the CFR was elaborated in each group and then it was extinguished for 6 days. Stressed rats of the STAND group showed the highest percentage of freezing time during the acquisition and extinction of CFR. The rats that were housed in the EE before receiving the restrictive stress froze to a less extent than the stressed rats of the STAND group. In other words, the 2-week pre-exposure of rats in the EE prevented the increase in CFR caused by the 2-h restrictive stress. It has been suggested that EE counteracts the strengthening of conditioned fear by increasing phosphorylation of the GluA1 subunit of AMPA receptors in the hippocampus induced by restrictive stress. In another study (Imanaka et al., 2006), the effect of EE (PNDs 22–49) on the contextual fear memory was studied in rats: (1) receiving neonatal isolation (PNDs 2–9) 1 h a day, (2) receiving single strong restrictive stress on PND 56 for 2 h followed by forced swimming for 20 min, (3) receiving neonatal isolation + stress, and (4) control animals. The control group, the rats subjected to severe single restrictive stress and stress combined with neonatal isolation, had the highest percentage of freezing time in the retention test 24 h after the CFR training. The EE rats showed a significant decrease in freezing time compared to the STAND group. The freezing time decreased substantially in the rats that received a severe single stress and a combination of a single stress and neonatal isolation. The EE impaired the CFR in these rats to the level of fear of the STAND group. Pavlova and Broshevitskaya (2021) studied the effect of EE (from 45 to 105 PNDs) on the contextual and cue fear memories in rats injected with a bacterial toxin, lipopolysaccharide, LPS on 3rd and 5th PNDs. Two other groups of animals were housed under SI and STAND conditions. The greatest attenuation of CFR was seen in rats subjected to SI and early life stress, both in males and females, in context and in response to CS. The EE caused a decrease of CFR during the retention test in the context compared to STAND conditions. Weakening of CFR also occurred when animals were transferred from SI to EE conditions (Mora-Gallegos and Fornaguera, 2019).

### The conditioned fear reaction in the presence of a conspecific partner

A lot of evidence testifies that EE through social communication with a conspecific partner has a beneficial effect on the organism. Staying together reduces the reaction to stress, alleviates anxiety, improves the process of wound healing, getting rid of other troubles and so on. This phenomenon, known as a “social buffer,” facilitates coping with the consequences of negative events (Glasper and Devries, 2005; Hennessy et al., 2009). It has been shown in experiments on rodents, that the presence of a conspecific partner reduces CFR and impairs the activity of the amygdalar complex in a dangerous environment and under the action of a cue stimulus (Kiyokawa et al., 2004, 2007; Fuzzo et al., 2015; Lee and Noh, 2016). Kiyokawa et al. (2004) divided the rats into two groups; one was the “experimental” (subject group) and the other - partner group. On the first day, the experimental rats and half of the partner rats learned the CFR (by use of 9 electroshocks, 55 V, 2 s) for 15 min with a random interval, while the other half of the partner rats did not learn the CFR, but the animals were placed in the same environment for 15 min. A total of 24 h later in the retention test the CFR of the experimental group were evaluated for 10 min, taking into account whether the rats were placed in the context one by one, together with a trained or untrained partner. The experimental rats froze the least time when they were in the context together with an untrained partner (about 100 s). The freezing time of rats tested alone was about 700 s, and tested together with a trained partner about 400 s. So, the partner rats significantly reduced the level of fear in experimental rats, acting as a social buffer (Kiyokawa et al., 2004). In another study (Fuzzo et al., 2015), rats were also exposed to CFR either alone or with a social partner placed on the other half of the chamber, divided in two parts by a transparent partition. After 24 h in the retention test, the rats that received CS-US pairings alone froze significantly longer than the rats being trained to CFR under conditions of visible social partner (170 s vs. 20 s, respectively). The presence of a conspecific partner significantly reduced the peak amplitude of auditory evoked potentials, gamma oscillations (25–75 Hz) and high-frequency oscillations (100–300 Hz) in the lateral amygdalar nucleus. The amplitude of the EP correlated with the duration of freezing response in rats trained to CFR (Fuzzo et al., 2015). In another study (Lee and Noh, 2016), in a chamber divided into two compartments, on one halve, the CFR was trained in rats placed singly or in two placed together at the same time. After 24 h in the retention test, the rats that learned the CFR together entered the dangerous compartment much earlier than those that were trained singly (about 70 and 150 s, respectively).

## Enriched environment and information processing

In the previous section, we have already marked the role of context and signal in the manifestations of CFR. Although memory represents the central core of a functional system, without the active work of the other key components of this system, the formation of memory, its retrieval (actualization) and consolidation is impossible. The information component, which includes the signal and the environmental stimuli (context), is an important part of the functional system which participates in the formation of sensory engrams of memory. Woodcock and Richardson (2000) conducted experiments on rats, which, from their point of view, reflect the speed and complicated character of the processing of sensory contextual information in rats housed in the EE and STAND environments. To study this, they used different times for rats to stay in the context (4, 16, and 120 s) before the onset of the electroshock. Rats were placed in the EE, starting from the 2nd PND until weaning from their mother (21st PND), and then continued to be housed in the EE until the 54–60th PNDs, before the start of behavioral experiments. The EE before weaning from a mother was induced by exposure to rat pups for 1 h with stimuli of various sensory modalities, and after weaning by placing them in a cage with tunnels, ladders, running wheels, etc. After exposure to the context for 4, 16, and 120 s, the rats received a single electroshock (0.5 mA, 1 s). 24 h later in the retention test, they were examined for the total freezing time. Being in the context of 4 and 120 s prior to the onset of the shock did not cause differences in the freezing time between the rats housed in the EE and STAND conditions, but the difference was significant at an interval of 16 s. The rats housed in the EE froze a significantly higher percentage of time than the rats of the STAND group (approximately 40% vs. 20%). On this basis, the authors concluded that the processing of contextual information passes faster in the EE group than in the STAND group. In their opinion, 4 s was insufficient time to process information about the environment in rats of both groups, while 120 s, on the contrary, was too long for both groups to process information about the context in full. The interval of 16 s was enough to discover the differential influence of the EE and STAND conditions on the processing of contextual information (Woodcock and Richardson, 2000). The faster information processing by rats of the EE group was testified by their ability to differentiate between neutral and “dangerous” contexts, whereas the STAND group could not do it. The increase of CFR in the EE rats could also be as a result of the sensitization of the fear system by the first stress produced by weaning rat pups from their mothers (Rosen and Schulkin, 1998). Clemenson et al. (2015) showed that pre-exposure of mice in context A for 10 min 24 h prior to CFR training caused different freezing times in contexts A and B 30 min after CFR acquisition. In control mice, the percentage of freezing time in the “dangerous” context A was higher than in the neutral context B. But the difference between the contexts disappeared in mice housed in the EE. Here again there was an important time given to mice for examining the context before switching on an electroshock. The mice of the EE group froze for a smaller percentage of time in context B than in context A at 5 and 15 s intervals, but the differences disappeared at an interval of 25 s (Clemenson et al., 2015).

We suppose that the correlation between the EE effects and strength of the conditioned fear association may be similar to the mechanisms of the latent inhibition. The latent inhibition is related with multiple pre-exposures of CS and CTX before CR learning. These pre-exposures slow down the acquisition of a conditioned reflex. It happens due to weakening of attention to the CS; a subject simply ignores it, considering it as an insignificant stimulus (Mackintosh, 1975).

Lubow et al. (1981) think that the slowdown in CR learning happens due to formation of a link between the repeated pre-exposures of future CS and no presentations of US. Because a future CS is always set up in a certain context, along with a loss of attention to CS, also takes place a loss of interest to the surrounding context. There are a number of hypotheses that explain the role of context in slowing down the development of conditioned associations. In particular, Lubow and Gewirtz (1995) consider context as an occasion-setter or as a “modulator” in a complex conditional stimulus. The signal (cue) and CTX are incorporated into this complex in such a way that, unreinforced presentations of CS and CTX form the CS-noUS and CTX-noUS associations before the development of CR. When CS-US pairings are presented in the same context where the CS pre-exposes (CS-noUS) and CTX pre-exposures (CTX-noUS) were used, then the context acquires a role of an occasion-setter (Fraser and Holland, 2019), which primarily affects the CS-noUS association and slows down a formation of a CS-US and CTX-US associations. It is possible that the mechanism of weakening the conditioned association by the EE is the same or similar as in latent inhibition. It is possible, that a long-term stay of animals in the EE creates similar to the effects of pre-exposures of CS and CTX, the strong connections of CS-noUS and CTX-noUS types, which in the future, during the CFR learning, significantly slow down and reduce fear, in contrast to the animals of the STAND group.

## Enriched environment and reinforcement (emotions)

If the signal and contextual stimuli, together with motivational excitation (will be discussed below), are associated with the formation and actualization of conditioned associations (sensory engrams), then the reinforcement function is more related with the consolidation of these associations. The hypothesis suggests that, in addition to the weakening of conditional associations during their formation under the influence of EE, there may also be a weakening of the consolidation of these associations as a result of a weakening of the reinforcement function. Many years ago, Fessler and Beatty (1976) discovered that rats housed in the EE (8–10 per cage), from 21 to 45 PND, showed higher thresholds of sensitivity to electroshock, than the animals of the STAND group. Both males and females had high thresholds for all behavioral responses (flinch, shuffle, and jump) caused by painful electrical stimulation. Later, this data was confirmed in other studies (Rocinholi et al., 1997; Cavalcante, 2019). Interestingly, only the 2-week, but not 4-week stay of rats in the EE caused an increase in thresholds and a decrease in the percentage of freezing time of animals in response to a 6-fold presentation of US during the learning phase. When tested after 24 h, CFR, in contrast to the unconditioned response, was approximately the same in the EE and STAND groups (Cavalcante, 2019). In a subsequent work (Cavalcante et al., 2020), the authors found an increase in dopaminergic neurons in the ventral tegmental area (VTA) under the influence of 2- and 4-weeks stay of animals in the EE. Coppens et al. (2010) suggested that EE affects dopamine release in the mesocorticolimbic pathways. Aumann et al. (2013) showed that the EE leads to an increase in the number of tyrosine hydroxylase immunoreactive neurons in the VTA and the nucleus accumbens. According to the authors, the housing conditions of animals affect the ratio of dopaminergic and non-dopaminergic neurons in VTA. The housing of rats for 6 weeks in the EE did not significantly influence the frequency of self-stimulation from electrodes located in the MFB near the VTA. However, the frequency of pedal pressing in this group tended to decrease (90 vs. 130), and the thresholds for frequency stimulation were higher (40 vs. 30 Hz) than in control animals (Konkle et al., 2010). It was shown that mice housed in the EE were less sensitive to the reinforcing effects of heroin, assessed by the conditioned place preference, than mice of the STAND group (El Rawas et al., 2009). Animals housed in the EE were less active on repeated injections of cocaine and less responsive to the cocaine challenge (Solinas et al., 2009). They were also less sensitive to the reinforcing effects of amphetamine, especially at low doses (Green et al., 2002). *In vivo* microdialysis, the positive effects of EE were independent of reduced dopamine levels in the ventral and dorsal striatum, but were associated with a reduction in the expression of the fast zif-268 gene in the nucleus accumbens.

Thus, the EE, on one hand, increases the threshold of sensitivity to the pain effects of electroshock, and on another hand, reduces the reinforcing properties of emotionally positive brain structures. These findings are consistent with our hypothesis that predicts a weakening of the brain’s reinforcing mechanisms responsible for consolidation of the conditioned fear memory in conditions of the EE.

## Enriched environment and motivation

The formation of CS-US associations requires the involvement of motivational mechanisms, which actively participate in the actualization of sensory engrams and triggering of the motor mechanisms for obtaining reinforcement. The hypothesis predicts that the EE should somehow impair the motivational state and ameliorate the actualization of S-S or S-R engrams (see Figure 1).

Barbelivien et al. (2006) in Exp. 3 measured the extinction of exploratory activity in a new context for 30 min in blocks of 5 min in the EE and STAND groups of rats. The decrease in total activity was more pronounced in the EE rats compared to the STAND group, starting from the 4th block. A comparison of exploratory activity within the EE group revealed a significant decrease in this activity already in the 2nd and the 3rd blocks. The authors suggested that the processing of sensory information in rats of the EE group occurs faster than in control rats. This accelerates the habituation of rats to the environment and the formation of indifference (familiarization) to the stimuli presented in it. These results have been confirmed in a number of other studies (Woodcock, 1994; Zimmermann et al., 2001; Schrijver et al., 2002). In particular, Schrijver et al. (2002) showed that rats housed in the EE had less exploratory activity in the object recognition test after moving the objects, than rats of the STAND group. In addition, they demonstrated a faster loss of interest in examining a new object by replacing an old one. In another experiment, the authors found a faster exit of rats of the EE group into the light section of the light-dark chamber, after the first entry into the dark section, compared with animals of the STAND group. The authors suggested that this occurs due to a faster examination of the dark compartment of the chamber by the EE group and habituation to it, similar to the behavior in the new object recognition test. Zimmermann et al. (2001) found that the EE group investigated more quickly, but also more quickly habituated to 4 new objects in an open field, than control animals. It was also shown that the EE mice better manifest a processing of new information than the STAND group, but the speed of information processing by animals of both groups did not depend on the complexity of this information (Cheng S. T. et al., 2022). Woodcock and Richardson (2000) measured the number of rats approaching to 12 focal points of an experimental chamber for 16 s preceding the electroshock. The EE rats did not differ from the STAND group either in the total number of focal points visited or in the number of visits to specific points. Since the EE rats froze significantly more time with this interval before shock presentation than the rats of the STAND group, the authors assumed that the EE group process contextual information faster than control animals. It should be noted that in a number of studies, the rats housed in the EE showed an increase in exploratory activity (Studelska and Kemble, 1979; Van Waas and Soffie, 1996; Brillaud et al., 2005; Pavlova et al., 2022, 2023). For example, in our work (Pavlova et al., 2022), males of the EE group traveled a greater distance and had a higher movement speed compared to males of the STAND group. They also had more hanging down in the elevated plus maze than males of the control group, while females of the EE group had more peeking into open arms. However, in these experiments, the EE procedure was short-term with placing the animals in it for 20 min, every second day. Van Waas and Soffie (1996), compared the effects of EE with standard conditions in young and old rats in models of spontaneous alternation and a new object recognition test, while Brillaud et al. (2005) in a radial-arm maze. In both studies, different results were obtained depending on the animal’s age and the models applied.

## Enriched environment and motor activity

Motor activity itself is one of the important components of the EE, since physical exercise (for example, running on a wheel) has a significant effect on plastic rearrangements in many brain structures. In particular, they have been shown to enhance the synaptic plasticity of the hippocampus (Patten et al., 2013), the density of dendritic spines (Dostes et al., 2016), the regulation of immediate fast genes (Simon et al., 2006), the activity of transcriptional genes (Chen and Russo-Neustadt, 2009) and trophic factors (Marlatt et al., 2012). This all leads to improved learning, better attention and spatial hippocampal-dependent memory. It is important at what age physical exercise starts. For example, in relation to the CFR, it was shown (O’Leary et al., 2019) that exercise in adult rats (8 weeks) for 7 weeks leads to an increase of conditioned fear in context and in response to a cue presentation. In adolescence (4 weeks), physical exercise did not affect the CFR, either in the context or on the cue, compared with control animals. Since the development of brain structures and functions continues during adolescence, one would expect that increased motor activity will produce more pronounced changes in CFR. However, physical exercise during adolescence did not affect the CFR. This could occur due to a number of specific molecular and cellular changes that are not manifested in adult animals (O’Leary et al., 2019). Specifically, these changes could be associated with increased activity of a number of genes that influence hippocampal plasticity, including genes for BDNF, synaptophysin, CREB, PSD-95, Arc, TLX, DCX, and others. Thus, physical exercise during the development of an organism through a sequence of molecular-cellular transformations may have a positive beneficial effect on the CFR manifestation. Concerning the motor activity in the EE, the hypothesis predicts its weakening or inadequate character (for example, impulsive behavior), since the motor component of the motor-reinforcing subsystem provides achievement of reinforcement and consolidation of CS-US associations.

Rats housed in the EE showed significantly lower spontaneous motor activity in the open field compared to the SI animals (Del Arco et al., 2007). Administration of the D1 agonist SKF38393 in the PFC produced long lasting increases in spontaneous motor activity but increases were significantly lower in the EE rats compared to the SI animals. Moreover, the density of D1 receptors in the PFC was significantly reduced in animals housed in the EE. Zambrana et al. (2007) showed that the EE animals spent less percentage of time in the open arms of an elevated plus maze and had much fewer entries than animals housed in the standard conditions. The authors suggested that the decreased percentage of time and number of entries in the open arms reflects a decreased novelty seeking in the EE mice, as a result of the abundant sensory stimulation in their housing conditions. EE has been shown to disrupt amphetamine-induced behavioral sensitization in motor activity without changing dopamine levels in the NAc and striatum (Bardo et al., 1995; Cheng C. N. et al., 2022). Mice raised in the EE showed less locomotor activity in response to an injection of cocaine than mice raised in a standard environment (Bezard et al., 2003). Sudo et al. (2018), in contrast, discovered that the EE enhances locomotor activity and leads to muscle hypertrophy without inducing physiological stress in rats. However, an increase in locomotor activity in the rats housed in the EE was observed only during the dark time of the day but not in the light time. The EE also affects impulsive behavior. Perry et al. (2008) showed that the EE rats had higher baseline MADs (a mean adjusted delay) (were less impulsive) than SI rats. In the study of Pattison et al. (2013), the pigeons were exposed to a version of the suboptimal choice task involving choice between an alternative that 50% of the time signaled 100% reinforcement, and 50% of the time signaled no food (the suboptimal alternative), and an alternative that 100% of the time signaled 75% reinforcement (the optimal alternative). The control group quickly showed a strong preference for the suboptimal alternative, whereas the EE group chose optimal for many training sessions, before eventually choosing the suboptimal alternative (Pattison et al., 2013; Zentall, 2021). In the experiments of Kirkpatrick et al. (2014), SI rats were more impulsive in delay-discounting schedule than the rats socially and sensory enriched, but they did not differ in risky choice behavior (a choice between a small constant reward versus large reward with low probability).

## Conclusion

In this review, a hypothesis explaining the positive (beneficial) effects of the EE on the CFR from the perspective of a functional behavioral control system is proposed. The central part in the functional system belongs to the memory apparatus, where the other key components of behavioral control (sensory information, motivation, motor actions and reinforcement) are linked. Memory represents the storage of sensory and motor engrams, which are formed and updated with the help of the sensory-motivational subsystem, while the other, motor-reinforcing subsystem controls the memory consolidation. Both subsystems interact through the inner functional circle of memory (see Figure 1). Housing in the EE changes the activity of all key components of the functional system in such a way that leads to a weakening of the CFR. First of all, the EE alters information processing, by promoting the fast habituation to the contextual stimuli and impairs incorporation (formation of sensory engrams) of these stimuli into the contextual memory. The weakening of the CS-US association under the effects of EE seems like the effects of latent inhibition. When animals are housed in the EE for a long time, similar to the effects of pre-exposures to CS and CTX at the latent inhibition, the associations of CS-noUS and CTX-noUS are formed. These [inhibitory] connections slow down the formation of CS-US and CTX-US associations and reduce the CFR, unlike animals of the STAND group, that have no EE experience. As in the case of CS pre-exposures (CS-noUS) during latent inhibition, the EE plays a role of the occasion-setter, primarily affecting the CS-noUS connection and, through it, promotes slowing down the formation of a CS-US association. The data collected in the literature shows that animals housed in the EE encounter a large amount of information, process and habituate to it faster. The fast information processing and its habituation, plus a weakening of the motivational mechanisms in the EE, impairs the CS-US associations (formation of sensory and motor engrams). The hypothesis predicts a weakening of the motivational mechanisms in the effects of the EE on CFR. It was shown in a number of tests (the open field, the elevated plus and radial-arm mazes, in the dark-light chamber, etc.) that EE promotes a more rapid decrease in exploratory activity and loss of interest in examining the environment compared with the control animals. The EE also affects the reinforcing brain mechanisms. According to the functional system of behavioral control, the reinforcement system plays an important role in consolidation of the CS-US associations. The EE impairs the reinforcing effects, which is proved by the increase of thresholds for pain sensitivity and self-stimulation, and decreases the sensitivity to the drugs of abuse (cocaine and amphetamine).

In conclusion, it should be noted that, of course, not all existing facts regarding the influence of the EE on the CFR are consistent with the predictions of the hypothesis. There are many reasons for this, which may be related to technical, procedural and methodological differences in the conduct of experiments. In recent years, a number of attempts have been made to create a more or less unified protocol for investigating the effects of an enriched environment on various forms of emotional and cognitive behavior (Harland and Dalrymple-Alford, 2020; Love et al., 2022). No doubt that it is very hard (if possible at all) to unify the characteristics of an enriched environment, the training methods (even within the same behavioral model), age, kind, genetic line of animals, etc. We did not aim in this review to consider a large number of variables affecting the EE influences on behavioral responses. But we tried to find a general (integrative) rule, a link between the influence of EE and the strength of the conditional (S-S) connection. The main outcome is that the EE has a beneficial effect on the fear response (weakens it). All other variables (like age of animal, type of enrichment, duration of stay in the EE, etc.) simply follow or should follow this rule. The hypothesis proposed and the available literature data on the example of a single neurobiological reaction, the CFR, provide conceptual justification for the influence of an enriched environment.

## Author contributions

The author confirms being the sole contributor of this work and has approved it for publication.

## References

[B1] Alarcón T. A. Presti-Silva S. M. Simões A. P. T. Ribeiro F. M. Pires R. G. W. (2023). Molecular mechanisms underlying the neuroprotection of environmental enrichment in Parkinson’s disease. *Neural. Regen. Res.* 18 1450–1456. 10.4103/1673-5374.360264 36571341 PMC10075132

[B2] Aronoff E. Hillyer R. Leon M. (2016). Environmental enrichment therapy for autism: Outcomes with increased access. *Neural. Plast.* 2020:2734915. 10.1155/2016/2734915 27721995 PMC5046013

[B3] Aumann T. D. Tomas D. Horne M. K. (2013). Environmental and behavioral modulation of the number of substantia nigra dopamine neurons in adult mice. *Brain Behav.* 3 617–625. 10.1002/brb3.163 24363965 PMC3868167

[B4] Ball N. J. Mercado E. III Orduña I. E. (2019). Enriched environments as a potential treatment for developmental disorders: Critical assessment. *Front. Psychol.* 10:466. 10.3389/fpsyg.2019.00466 30894830 PMC6414413

[B5] Barbelivien A. Herbeaux K. Oberling P. Kelche C. Galani R. Majchrzak M. (2006). Environmental enrichment increases responding to contextual cues but decreases overall conditioned fear in the rat. *Behav. Brain Res.* 169 231–238. 10.1016/j.bbr.2006.01.012 16473418

[B6] Bardo M. T. Bowling S. L. Rowlett J. K. Manderscheid P. Buxton S. T. Dwoskin L. P. (1995). Environmental enrichment attenuates locomotor sensitization, but not in vitro dopamine release, induced by amphetamine. *Pharmacol. Biochem. Behav.* 51 397–405. 10.1016/0091-3057(94)00413-d 7667360

[B7] Benaroya-Milshtein N. Hollander N. Apter A. Kukulansky T. Raz N. Wilf A. (2004). Environmental enrichment in mice decreases anxiety, attenuates stress responses and enhances natural killer cell activity. *Eur. J. Neurosci.* 20 1341–1347. 10.1111/j.1460-9568.2004.03587.x 15341605

[B8] Bezard E. Dovero S. Belin D. Duconger S. Jackson-Lewis V. Przedborski S. (2003). Enriched environment confers resistance to 1-methyl-4- phenyl-1,2,3,6-tetrahydropyridine and cocaine: Involvement of dopamine transporter and trophic factors. *J. Neurosci.* 23 10999–11007. 10.1523/JNEUROSCI.23-35-10999.2003 14657156 PMC6741042

[B9] Brenes J. C. Lackinger M. Höglinger G. U. Schratt G. Schwarting R. K. Wöhr M. (2016). Differential effects of social and physical environmental enrichment on brain plasticity, cognition, and ultrasonic communication in rats. *J. Comp. Neurol.* 524 1586–1607. 10.1002/cne.23842 26132842

[B10] Brillaud E. Morillion D. de Seze R. (2005). Modest environmental enrichment: Effect on a radial maze validation and well being of rats. *Brain Res.* 1054 174–182. 10.1016/j.brainres.2005.06.069 16098485

[B11] Campeau S. Nyhuis T. J. Sasse S. K. Kryskow E. M. Herlihy L. Masini C. V. (2010). Hypothalamic pituitary adrenal axis responses to low-intensity stressors are reduced after voluntary wheel running in rats. *J. Neuroendocrinol.* 22 872–888. 10.1111/j.1365-2826.2010.02007.x 20406350 PMC4469265

[B12] Cavalcante K. M. H. (2019). Short-term but not long-term exposure to an enriched environment reduces unconditioned fear responses but not conditioned fear responses. *Sci. Electron. Arch.* 12 86–94. 10.36560/1252019933

[B13] Cavalcante K. M. H. Bispo J. M. M. Souza M. F. Medeiros K. A. A. L. Lins L. C. R. F. Santos E. R. (2020). Short-term but not long-term exposure to an enriched environment facilitates the extinction of aversive memory. *Behav. Brain Res.* 393:112806. 10.1016/j.bbr.2020.112806 32673706

[B14] Chen M. J. Russo-Neustadt A. A. (2009). Running exercise-induced up-regulation of hippocampal brain-derived neurotrophic factor is CREB-dependent. *Hippocampus* 19 962–972. 10.1002/hipo.20579 19294650 PMC2756465

[B15] Cheng C. N. Wu S. J. Huang A. C. W. (2022). Environmental enrichment components required to reduce methamphetamine-induced behavioral sensitization in mice: Examination of behaviors and neural substrates. *J. Clin. Med.* 11:3051. 10.3390/jcm11113051 35683439 PMC9181252

[B16] Cheng S. T. Liu S. Ou-Yang B. Dai X. Y. Cheng L. (2022). Specific effects of characteristics of enriched environment on innovative problem solving by animals. *Psychol. Sci.* 33 1097–1111. 10.1177/09567976211070562 35776087

[B17] Clemenson G. D. Lee S. W. Deng W. Barrera V. R. Iwamoto K. S. Fanselow M. S. (2015). Enrichment rescues contextual discrimination deficit associated with immediate shock. *Hippocampus* 25 385–392. 10.1002/hipo.22380 25330953 PMC4398310

[B18] Coppens C. M. de Boer S. F. Koolhaas J. M. (2010). Coping styles and behavioural flexibility: Towards underlying mechanisms. *Philos. Trans. R. Soc. Lond. B. Biol. Sci.* 365 4021–4028. 21078654 10.1098/rstb.2010.0217 PMC2992750

[B19] Costa-López B. Ferrer-Cascales R. Ruiz-Robledillo N. Albaladejo-Blázquez N. Baryła-Matejczuk M. (2021). Relationship between sensory processing and quality of life: A systematic review. *J. Clin. Med.* 10:3961. 10.3390/jcm10173961 34501408 PMC8432132

[B20] Colavitta M. F. Grasso L. Barrantes F. J. (2023). Environmental enrichment in murine models and its translation to human factors improving conditions in Alzheimer disease. *J. Prev. Alzheimers Dis.* 10 287–300. 10.14283/jpad.2023.5 36946456

[B21] Cummins R. A. Livesey P. J. Evans J. G. (1977). A developmental theory of environmental enrichment. *Science* 197 692–694. 10.1126/science.877587 877587

[B22] Davim A. Trindade da Silva L. Vieira P. (2021). Environmental enrichment as a strategy to confront social isolation under the COVID-19 pandemic. *Front. Behav. Neurosci.* 14:564184. 10.3389/fnbeh.2020.564184 33551762 PMC7859510

[B23] Del Arco A. Segovia G. Canales J. J. Garrido P. de Blas M. García-Verdugo J. M. (2007). Environmental enrichment reduces the function of D1 dopamine receptors in the prefrontal cortex of the rat. *J. Neural. Transm.* 114 43–48. 10.1007/s00702-006-0565-8 16955373

[B24] Dostes S. Dubreucq S. Ladevèze E. Marsicano G. Abrous D. N. Chaouloff F. (2016). Running per se stimulates the dendritic arbor of newborn dentate granule cells in mouse hippocampus in a duration-dependent manner. *Hippocampus.* 26 282–288. 10.1002/hipo.22551 26606164

[B25] Duffy S. N. Craddock K. J. Abel T. Nguyen P. V. (2001). Environmental enrichment modifies the PKA-dependence of hippocampal LTP and improves hippocampus-dependent memory. *Learn. Mem.* 8 26–34. 10.1101/lm.36301 11160761 PMC311356

[B26] Duman C. H. Schlesinger L. Russell D. S. Duman R. S. (2008). Voluntary exercise produces antidepressant and anxiolytic behavioral effects in mice. *Brain Res.* 1199 148–158. 10.1016/j.brainres.2007.12.047 18267317 PMC2330082

[B27] El Rawas R. Thiriet N. Lardeux V. Jaber M. Solinas M. (2009). Environmental enrichment decreases the rewarding but not the activating effects of heroin. *Psychopharmacology* 203 561–570. 10.1007/s00213-008-1402-6 19005643

[B28] Escobar M. Oberling P. Miller R. R. (2002). Associative deficit accounts of disrupted latent inhibition and blocking in schizophrenia. *Neurosci. Biobehav. Rev.* 26 203–216. 10.1016/s0149-7634(01)00067-7 11856559

[B29] Fessler R. G. Beatty W. W. (1976). Variations in postweaning environment and sensitivity to electric shock in male and female rats. *Behav. Biol.* 16 535–538. 10.1016/s0091-6773(76)91747-8 962771

[B30] Forster B. Eardley A. F. Eimer M. (2007). Altered tactile spatial attention in the early blind. *Brain Res.* 1131 149–154. 10.1016/j.brainres.2006.11.004 17173872

[B31] Fraser K. M. Holland P. C. (2019). Occasion setting. *Behav. Neurosci.* 133 145–175. 10.1037/bne0000306 30907616 PMC6447318

[B32] Fuzzo F. Matsumoto J. Kiyokawa Y. Takeuchi Y. Ono T. Nishijo H. (2015). Social buffering suppresses fear-associated activation of the lateral amygdala in male rats: Behavioral and neurophysiological evidence. *Front. Neurosci.* 9:99. 10.3389/fnins.2015.00099 25859179 PMC4373252

[B33] Glasper E. R. Devries A. C. (2005). Social structure influences effects of pair-housing on wound healing. *Brain Behav. Immun.* 19 61–68. 10.1016/j.bbi.2004.03.002 15581739

[B34] Grahame N. J. Barnet R. C. Gunther L. M. Miller R. R. (1994). Latent inhibition as a performance deficit resulting from CS-context associations. *Anim. Learn. Behav.* 22 395–408. 10.3758/BF03209159

[B35] Gray J. A. (1982). Précis of the neuropsychology of anxiety: An enquiry into the functions of the septo-hippocampal system. *Behav. Brain Sci.* 5 469–484.

[B36] Green T. Gehrke B. Bardo M. (2002). Environmental enrichment decreases intravenous amphetamine self-administration in rats: Dose-response functions for fixed- and progressive-ratio schedules. *Psychopharmacology* 162 373–378. 10.1007/s00213-002-1134-y 12172690

[B37] Grigoryan G. A. (1990). Interaction of signal, motivational and executive components of conditioned reflex. *Zh. Visch. Nervn. Deyat.* 40 629–642. 2174604

[B38] Grigoryan G. A. (2006). Memory and depression. *Zh. Visch. Nervn. Deyat*. 56 556–570.

[B39] Grigoryan G. A. (2021). Molecular-cellular mechanisms of plastic restructuring produced by an enriched environment. Effects on learning and memory. *Neurochem. J.* 15 226–239. 10.1134/S1819712421030041

[B40] Grigoryan G. A. Gulyaeva N. V. (2017). Modeling depression in animals: Behavior as the basis for the methodology, assessment criteria, and classification. *Neurosci. Behav. Physiol.* 47 204–216. 10.1007/s11055-016-0386-7

[B41] Grigoryan G. A. Markevich V. A. (2015). Consolidation, reactivation, and reconsolidation of memory. *Neurosci. Behav. Physiol.* 45 1019–1028. 10.1007/s11055-015-0181-x

[B42] Guan S. Z. Fu Y. J. Zhao F. Liu H. Y. Chen X. H. Qi F. Q. (2021). The mechanism of enriched environment repairing the learning and memory impairment in offspring of prenatal stress by regulating the expression of activity-regulated cytoskeletal-associated and insulin-like growth factor-2 in hippocampus. *Environ. Health Prev. Med.* 26:8. 10.1186/s12199-020-00929-7 33451279 PMC7811238

[B43] Hannan A. J. (2014). Environmental enrichment and brain repair: Harnessing the therapeutic effects of cognitive stimulation and physical activity to enhance experience-dependent plasticity. *Neuropathol. Appl. Neurobiol.* 40 13–25. 10.1111/nan.12102 24354721

[B44] Harland B. C. Dalrymple-Alford J. C. (2020). Enriched environment procedures for rodents: Creating a standardized protocol for diverse enrichment to improve consistency across research studies. *Bio Protoc.* 10:e3637. 10.21769/BioProtoc.3637 33659308 PMC7842323

[B45] Hegde P. O’Mara S. Laxmi T. R. (2017). Extinction of contextual fear with timed exposure to enriched environment: A differential effect. *Ann. Neurosci.* 24 90–104. 10.1159/000475898 28588364 PMC5448453

[B46] Hendriksen H. Prins J. Olivier B. Oosting R. S. (2010). Environmental enrichment induces behavioral recovery and enhanced hippocampal cell proliferation in an antidepressant-resistant animal model for PTSD. *PLoS One* 5:e11943. 10.1371/journal.pone.0011943 20700523 PMC2916817

[B47] Hennessy M. B. Kaiser S. Sachser N. (2009). Social buffering of the stress response: Diversity, mechanisms, and functions. *Front. Neuroendocrinol.* 30:470–482. 10.1016/j.yfrne.2009.06.001 19545584

[B48] Hunter A. S. (2015). Impaired extinction of fear conditioning after REM deprivation is magnified by rearing in an enriched environment. *Neurobiol. Learn. Mem.* 122 11–18. 10.1016/j.nlm.2015.01.003 25602928

[B49] Imanaka A. Morinobu S. Toki S. Yamawaki S. (2006). Importance of early environment in the development of post-traumatic stress disorder-like behaviors. *Behav. Brain Res.* 173 129–137. 10.1016/j.bbr.2006.06.012 16860405

[B50] Ji N. N. Jiang H. Xia M. (2022). The influence of the enriched environment in different periods on neonatal maternal separation-induced visceral pain, anxiousness, and depressive behaviors. *Transl. Pediatr.* 11 1562–1569. 10.21037/tp-22-475 36247898 PMC9561516

[B51] Jungling A. Reglodi D. Karadi Z. N. Horvath G. Farkas J. Gaszner B. (2017). Effects of postnatal enriched environment in a model of Parkinson’s disease in adult rats. *Int. J. Mol. Sci*. 18:406. 10.3390/ijms18020406 28216584 PMC5343940

[B52] Kempermann G. Kuhn H. G. Gage F. H. (1997). More hippocampal neurons in adult mice living in an enriched environment. *Nature* 386 493–495. 10.1038/386493a0 9087407

[B53] Kirkpatrick K. Marshall A. T. Smith A. P. Koci J. Park Y. (2014). Individual differences in impulsive and risky choice: Effects of environmental rearing conditions. *Behav. Brain Res.* 269 115–127. 10.1016/j.bbr.2014.04.024 24769268 PMC4069030

[B54] Kiyokawa Y. Kikusui T. Takeuchi Y. Mori Y. (2004). Partner’s stress status influences social buffering effects in rats. *Behav. Neurosci.* 118 798–804. 10.1037/0735-7044.118.4.798 15301605

[B55] Kiyokawa Y. Takeuchi Y. Mori Y. (2007). Two types of social buffering differentially mitigate conditioned fear responses. *Eur. J. Neurosci.* 26 3606–3613. 10.1111/j.1460-9568.2007.05969.x 18052972

[B56] Konkle A. T. Kentner A. C. Baker S. L. Stewart A. Bielajew C. (2010). Environmental-enrichment-related variations in behavioral, biochemical, and physiologic responses of Sprague-Dawley and Long Evans rats. *J. Am. Assoc. Lab. Anim. Sci.* 49 427–436. 20819388 PMC2919182

[B57] Lach G. Bicca M. A. Hoeller A. A. Santos E. C. Costa A. P. de Lima T. C. (2016). Short-term enriched environment exposure facilitates fear extinction in adult rats: The NPY-Y1 receptor modulation. *Neuropeptides* 55 73–78. 10.1016/j.npep.2015.10.001 26490304

[B58] Laviola G. Hannan A. J. Macrì S. Solinas M. Jaber M. (2008). Effects of enriched environment on animal models of neurodegenerative diseases and psychiatric disorders. *Neurobiol. Dis.* 31 159–168. 10.1016/j.nbd.2008.05.001 18585920

[B59] Lee H. Noh J. (2016). Pair exposure with conspecific during fear conditioning induces the link between freezing and passive avoidance behaviors in rats. *Neurosci. Res.* 108 40–45. 10.1016/j.neures.2016.01.005 26827818

[B60] Leger M. Paizanis E. Dzahini K. Quiedeville A. Bouet V. Cassel J. C. (2015). Environmental enrichment duration differentially affects behavior and neuroplasticity in adult mice. *Cereb. Cortex* 25 4048–4061. 10.1093/cercor/bhu119 24904072

[B61] Leon M. Woo C. (2018). Environmental enrichment and successful aging. *Front. Behav. Neurosci.* 12:155. 10.3389/fnbeh.2018.00155 30083097 PMC6065351

[B62] Lewis D. J. (1979). Psychobiology of active and inactive memory. *Psychol. Bull.* 86 1054–1083. 386401

[B63] Li J. Z. Hao X. H. Wu H. P. Li M. Liu X. M. Wu Z. B. (2021). An enriched environment delays the progression from mild cognitive impairment to Alzheimer’s disease in senescence-accelerated mouse prone 8 mice. *Exp. Ther. Med.* 22:1320. 10.3892/etm.2021.10755 34630674 PMC8495563

[B64] Liew A. K. Y. Teo C. H. Soga T. (2022). The molecular effects of environmental enrichment on Alzheimer’s disease. *Mol. Neurobiol.* 59 7095–7118. 10.1007/s12035-022-03016-w 36083518 PMC9616781

[B65] Love C. J. Gubert C. Renoir T. Hannan A. J. (2022). Environmental enrichment and exercise housing protocols for mice. *STAR Protoc.* 3:101689. 10.1016/j.xpro.2022.101689 36125931 PMC9493135

[B66] Lubow R. E. Gewirtz J. C. (1995). Latent inhibition in humans: Data, theory, and implications for schizophrenia. *Psychol. Bull.* 117 87–103. 10.1037/0033-2909.117.1.87 7870865

[B67] Lubow R. E. Weiner I. Schnur P. (1981). “Conditioned attention theory,” in *The psychology of learning and motivation*, Vol. 15 ed. Bower G. H. (New York, NY: Academic Press), 1–49.

[B68] Mackintosh N. J. (1975). The theory of attention: Variation in associability of stimuli with reinforcement. *Psychol. Rev.* 82 276–298. 10.1037/h0076778

[B69] Marlatt M. W. Potter M. C. Lucassen P. J. van Praag H. (2012). Running throughout middle-age improves memory function, hippocampal neurogenesis, and BDNF levels in female C57BL/6J mice. *Dev. Neurobiol.* 72 943–952. 10.1002/dneu.22009 22252978 PMC3485396

[B70] Mora-Gallegos A. Fornaguera J. (2019). The effects of environmental enrichment and social isolation and their reversion on anxiety and fear conditioning. *Behav. Process.* 158 59–69. 10.1016/j.beproc.2018.10.022 30389595

[B71] Nader K. Schafe G. E. LeDoux J. E. (2000). Fear memories require protein synthesis in the amygdala for reconsolidation after retrieval. *Nature* 406 722–726. 10963596 10.1038/35021052

[B72] Nejati V. (2018). Effects of sensory deprivation on cognitive degeneration: Evidence from ageing individuals with blindness. *Neurol. Psychiatry Brain Res.* 27 27–31. 10.1016/j.npbr.2017.12.004

[B73] Novaes L. S. Bueno-de-Camargo L. M. Munhoz C. D. (2021). Environmental enrichment prevents the late effect of acute stress-induced fear extinction deficit: The role of hippocampal AMPA-GluA1 phosphorylation. *Transl. Psychiatry* 11:18. 10.1038/s41398-020-01140-6 33414437 PMC7791025

[B74] Novati A. Nguyen H. P. Schulze-Hentrich J. (2022). Environmental stimulation in Huntington disease patients and animal models. *Neurobiol. Dis.* 171:105725. 10.1016/j.nbd.2022.105725 35427742

[B75] O’Leary J. D. Hoban A. E. Cryan J. F. O’Leary O. F. Nolan Y. M. (2019). Differential effects of adolescent and adult-initiated voluntary exercise on context and cued fear conditioning. *Neuropharmacology* 145(Pt. A) 49–58. 10.1016/j.neuropharm.2018.05.007 29793890

[B76] Patten A. R. Sickmann H. Hryciw B. N. Kucharsky T. Parton R. Kernick A. (2013). Long-term exercise is needed to enhance synaptic plasticity in the hippocampus. *Learn. Mem.* 20 642–647. 10.1101/lm.030635.113 24131795

[B77] Pattison K. F. Laude J. R. Zentall T. R. (2013). Social enrichment affects suboptimal, risky, gambling-like choice by pigeons. *Anim. Cog.* 16 429–434. 10.1007/s10071-012-0583-x 23224431 PMC3628401

[B78] Pavlova I. V. Broshevitskaya N. D. (2021). The Influence of social isolation and enriched environment on fear conditioning in rats after early proinflammatory stress. *J. Evol. Bioch. Physiol.* 57 803–816. 10.1134/s0022093021040062

[B79] Pavlova I. V. Broshevitskaya N. D. Zaichenko M. I. Grigoryan G. A. (2022). Effects of social isolation and an enriched environment on anxious-depressive behavior in rats in normal conditions and after early proinflammatory stress. *Neurosci. Behav. Physiol.* 52 684–697. 10.1007/s11055-022-01294-4

[B80] Pavlova I. V. Broshevitskaya N. Zaichenko M. I. Grigoryan G. A. (2023). The influence of long-term housing in enriched environment on behavior of normal rats and subjected to early pro-inflammatory lipopolysaccharide stress. *Brain Behav. Immun. Health* 30:100639. 10.1016/j.bbih.2023.100639 37274935 PMC10236189

[B81] Perry J. L. Stairs D. J. Bardo M. T. (2008). Impulsive choice and environmental enrichment: Effects of d-amphetamine and methylphenidate. *Behav. Brain Res.* 193 48–54. 10.1016/j.bbr.2008.04.019 18534693 PMC2681296

[B82] Pietropaolo S. Feldon J. Yee B. K. (2014). Environmental enrichment eliminates the anxiety phenotypes in a triple transgenic mouse model of Alzheimer’s disease. *Cognit. Affect Behav. Neurosci.* 14 996–1008. 10.3758/s13415-014-0253-3 24492993

[B83] Rabadán R. Ramos-Campos M. Rosa Redolat R. Mesa-Gresa P. (2019). Physical activity and environmental enrichment: Behavioural effects of exposure to different housing conditions in mice. *Acta Neurobiol. Exp.* 79 374–385. 10.21307/ane-2019-035 31885394

[B84] Rampon C. Tang Y.-P. Goodhouse J. Shimizu E. Kyin M. Tsien J. Z. (2000). Enrichment induced structural changes and recovery from nonspatial memory deficits in CA1 NMDAR1-knockout mice. *Nat. Neurosci.* 3 238–245. 10.1038/72945 10700255

[B85] Rescorla R. A. Wagner A. R. (1972). “A theory of Pavlovian conditioning; variations in the effectiveness of reinforcement and nonreinforcement,” in *Classical conditioning II: Current research and theory*, eds Black A. H. Prosaky W. F. (New York, NY: Appleton-Century-Crofts), 64–99.

[B86] Rocinholi L. F. Almeida S. S. De-Oliveira L. M. (1997). Response threshold to aversive stimuli in stimulated early protein-malnourished rats. *Braz. J. Med. Biol. Res.* 30 407–413. 10.1590/s0100-879x1997000300016 9246240

[B87] Rosen J. B. Schulkin J. (1998). From normal fear to pathological anxiety. *Psychol. Rev*. 105 325–350. 10.1037/0033-295x.105.2.325 9577241

[B88] Rosenzweig M. R. Bennett E. L. Hebert M. Morimoto H. (1978). Social grouping cannot account for cerebral effects of enriched environments. *Brain Res.* 153 563–576. 10.1016/0006-8993(78)90340-2 698794

[B89] Sale A. Berardi N. Maffei L. (2009). Enrich the environment to empower the brain. *Trends Neurosci.* 32 233–239. 10.1016/j.tins.2008.12.004 19268375

[B90] Schrijver N. C. A. Bahr N. I. Weiss I. C. Würbel H. (2002). Dissociable effects of isolation rearing and environmental enrichment on exploration, spatial learning and HPA activity in adult rats. *Pharm. Biochem. Behav.* 73 209–224. 10.1016/s0091-3057(02)00790-6 12076740

[B91] Schroyens N. Beckers T. Luyten L. (2023). Appraising reconsolidation theory and its empirical validation. *Psychon. Bull. Rev.* 30 450–463. 10.3758/s13423-022-02173-2 36085236 PMC7614440

[B92] Simon P. Fehrenbach E. Niess A. M. (2006). Regulation of immediate early gene expression by exercise: Short cuts for the adaptation of immune function. *Exerc. Immunol. Rev.* 12 112–131. 17201076

[B93] Smail M. A. Smith B. L. Nawreen N. Herman J. P. (2020). Differential impact of stress and environmental enrichment on corticolimbic circuits. *Pharmacol. Biochem. Behav.* 197:172993. 10.1016/j.pbb.2020.172993 32659243 PMC7484282

[B94] Solinas M. Thiriet N. El Rawas R. Lardeux V. Jaber M. (2009). Environmental enrichment during early stages of life reduces the behavioral, neurochemical, and molecular effects of cocaine. *Neuropsychopharmacology* 34 1102–1111. 10.1038/npp.2008.51 18463628

[B95] Sommerlad A. Sabia S. Singh-Manoux A. Lewis G. Livingston G. (2019). Association of social contact with dementia and cognition: 28-year follow-up of the Whitehall II cohort study. *PLoS Med.* 16:e1002862. 10.1371/journal.pmed.1002862 31374073 PMC6677303

[B96] Spires T. L. Grote H. E. Varshney N. K. Cordery P. M. van Dellen A. Blakemore C. (2004). Environmental enrichment rescues protein deficits in a mouse model of Huntington’s disease, indicating a possible disease mechanism. *J. Neurosci.* 24 2270–2276. 10.1523/JNEUROSCI.1658-03.2004 14999077 PMC6730435

[B97] Studelska D. R. Kemble E. D. (1979). Effects of briefly experienced environmental complexity on openfield behavior in rats. *Behav. Neural Biol.* 26 492–496. 10.1016/S0163-1047(79)91554-1

[B98] Sudo M. Nagamatsu T. Ando S. (2018). Does environmental enrichment increase locomotor activity in rats? Evidence from an implanted sensor device. *Bull. Phys. Fitness Res. Inst.* 16 29–32.

[B99] Sukegawa M. Yoshihara T. Hou S. Asano M. Hannan A. J. Wang D. O. (2022). Longlasting housing environment manipulation and acute loss of environmental enrichment impact BALB/c mice behaviour in multiple functional domains. *Eur. J. Neurosci.* 55 1118–1140. 10.1111/ejn.15602 35060219 PMC9306724

[B100] Sun X. R. Zhang H. Zhao H. T. Ji M. H. Li H. H. Wu J. (2016). Amelioration of oxidative stress-induced phenotype loss of parvalbumin interneurons might contribute to the beneficial effects of environmental enrichment in a rat model of post-traumatic stress disorder. *Behav. Brain Res.* 312 84–92. 10.1016/j.bbr.2016.06.016 27297027

[B101] Takahashi T. Shimizu K. Shimazaki K. Toda H. Nibuya M. (2014). Environmental enrichment enhances autophagy signaling in the rat hippocampus. *Brain Res.* 1592 113–123. 10.1016/j.brainres.2014.10.026 25451096

[B102] Tang Y.-P. Wang H. Feng R. Kyin M. Tsien J. Z. (2001). Differential effects of enrichment on learning and memory function in NR2B transgenic mice. *Neuropharmacology* 41 779–790. 10.1016/s0028-3908(01)00122-8 11640933

[B103] Tanichi M. Toda H. Shimizu K. Koga M. Saito T. Enomoto S. (2018). Differential effects of voluntary wheel running and toy rotation on the mRNA expression of neurotrophic factors and FKBP5 in a post-traumatic stress disorder rat model with the shuttle-box task. *Biochem. Biophys. Res. Commun.* 501 307–312. 10.1016/j.bbrc.2018.05.023 29738768

[B104] Van Praag H. Kempermann G. Gage F. H. (2000). Neural consequences of environmental enrichment. *Nat. Rev. Neurosci.* 1 191–198. 10.1038/35044558 11257907

[B105] Van Waas M. Soffie M. (1996). Differential environmental modulations on locomotor activity, exploration and spatial behaviour in young and old rats. *Physiol. Behav.* 59 265–271. 10.1016/0031-9384(95)02151-5 8838604

[B106] Vecchi T. Girelli L. (1998). Gender differences in visuo-spatial processing: The importance of distinguishing between passive storage and active manipulation. *Acta Psychol.* 99 1–16. 10.1016/s0001-6918(97)00052-8 9664836

[B107] Woodcock E. A. (1994). *The enriched environment as a tool for investigating environmentally-mediated behavioural and neural plasticity. Unpublished Honors’ Thesis.* Sydney, NSW: University of New South Wales.

[B108] Woodcock E. A. Richardson R. (2000). Effects of environmental enrichment on rate of contextual processing and discriminative ability in adult rats. *Neurobiol. Learn. Mem.* 73 1–10. 10.1006/nlme.1999.3911 10686119

[B109] Xu H. Li B. Li L. Fan Z. Gong X. Wu L. (2022). Environmental enrichment mitigates PTSD-like behaviors in adult male rats exposed to early life stress by regulating histone acetylation in the hippocampus and amygdala. *J. Psychiatr. Res.* 155 120–136. 10.1016/j.jpsychires.2022.07.067 36029624

[B110] Yu Y. H. Lim Y. S. Ou C. Y. Chang K. C. Tsai A. C. Chang F. C. (2022). The medial prefrontal cortex, nucleus accumbens, basolateral amygdala, and hippocampus regulate the amelioration of environmental enrichment and cue in fear behavior in the animal model of PTSD. *Behav. Neurol.* 2022:7331714. 10.1155/2022/7331714 35178125 PMC8843982

[B111] Zambrana C. Marco E. M. Arranz L. de Castro N. M. Viveros M. P. de la Fuente M. (2007). Influence of aging and enriched environment on motor activity and emotional responses in mice. *Ann. N. Y. Acad. Sci.* 1100 543–552. 10.1196/annals.1395.060 17460220

[B112] Zentall T. R. (2021). Effect of environmental enrichment on the brain and on learning and cognition by animals. *Animals* 11:973. 10.3390/ani11040973 33807367 PMC8066627

[B113] Zhang Y. M. Cheng Y. Z. Wang Y. T. Wei R. M. Ge Y. J. Kong X. Y. (2022). Environmental enrichment reverses maternal sleep deprivation-induced anxiety-like behavior and cognitive impairment in CD-1 mice. *Front. Behav. Neurosci.* 16:943900. 10.3389/fnbeh.2022.943900 35910680 PMC9326347

[B114] Zimmermann A. Stauffacher M. Langhans W. Würbel H. (2001). Enrichment-dependent differences in novelty exploration in rats can be explained by habituation. *Behav Brain Res.* 121 11–20. 11275280 10.1016/s0166-4328(00)00377-6

